# Graphene based room temperature flexible nanocomposites from permanently cross-linked networks

**DOI:** 10.1038/s41598-018-21114-5

**Published:** 2018-02-12

**Authors:** Nishar Hameed, Ludovic F. Dumée, Francois-Marie Allioux, Mojdeh Reghat, Jeffrey S. Church, Minoo Naebe, Kevin Magniez, Jyotishkumar Parameswaranpillai, Bronwyn L. Fox

**Affiliations:** 1 Factory of the Future, Swinburne University of Technology, Hawthorn, Australia; 2 Deakin University, Geelong, Institute for Frontier Materials, Waurn Ponds, Victoria, 3216 Australia; 3 CSIRO Manufacturing, Waurn Ponds, Victoria, 3216 Australia; 4 Department of Polymer Science and Rubber Technology, Cochin University of Science and Technology, Cochin, India

## Abstract

Graphene based room temperature flexible nanocomposites were prepared using epoxy thermosets for the first time. Flexible behavior was induced into the epoxy thermosets by introducing charge transfer complexes between functional groups within cross linked epoxy and room temperature ionic liquid ions. The graphene nanoplatelets were found to be highly dispersed in the epoxy matrix due to ionic liquid cation–π interactions. It was observed that incorporation of small amounts of graphene into the epoxy matrix significantly enhanced the mechanical properties of the epoxy. In particular, a 0.6 wt% addition increased the tensile strength and Young’s modulus by 125% and 21% respectively. The electrical resistance of nanocomposites was found to be increased with graphene loading indicating the level of self-organization between the ILs and the graphene sheets in the matrix of the composite. The graphene nanocomposites were flexible and behave like ductile thermoplastics at room temperature. This study demonstrates the use of ionic liquid as a compatible agent to induce flexibility in inherently brittle thermoset materials and improve the dispersion of graphene to create high performance nanocomposite materials.

## Introduction

There has recently been great interest in making flexible/bendable electronics such as rollup displays and wearable devices with graphene based polymer composites typically being used as a flexible substrate^1,2^. However, a major challenge in the fabrication of such flexible devices is the unavailability of suitable materials that combine high conductivity, mechanical flexibility and stability in electrochemical environments. Furthermore, most of the commonly used polymer matrices rapidly degrade at relatively low temperature^3^. The use of permanently cross-linked polymer structures such as epoxy thermosets could potentially offer high mechanical properties and stability^4^. They have high dimensional stability, high mechanical, thermal, and environmental resistance; and are therefore utilized in many challenging applications^5^.

Thermoset epoxies are however highly brittle materials and thus offer no or limited flexibility. There are a few reports that focused on the formation of cross-linked networks that can disrupt and reform^6,7^ based on addition-fragmentation chain transfer reactions in the presence of radicals^8,9^. However, such networks show limited reversibility and the chemical equilibrium can be displaced towards depolymerisation, leading to unfavorable network structure and performance^10^. Leibler *et al*. reported the synthesis of reversible epoxy networks based on exchange reactions that lead to thermoset materials with high levels of stress relaxation and recyclability^11^. This approach was further extended to other olefin containing polymers^12^.

Here we report an epoxy/graphene nanocomposite that is flexible and deformable after cross-linking and behaves like ductile thermoplastics. The flexibility was induced by the introduction of an imidazolium based room temperature ionic liquid. Structurally, ILs are molecular ions containing a bulky organic cation and a small anion^13^. Here, ILs act as miscible molecular liquids to form charge transfer complexes with the epoxy and thus become part of the cross-linked network giving the materials distinctive thermo-mechanical properties^14^. Moreover, ILs are also known to possess excellent graphene dispersion capabilities^15^. Imidazolium based ILs have been used to make stable dispersions of reduced graphene oxide at relatively high concentration without using any surfactants/stabilizers. In this work, epoxy based flexible graphene nanocomposites were successfully prepared and characterized for their use in functional applications.

## Experimental

Graphene nanoplatelets were obtained from XG Sciences commonly known as xGnP^®^ graphene nanoplatelets. The platelets have an average dimeter of 5 micron and a typical surface area of 120–150 m^2^.g^−1^. The epoxy precursor diglycidyl ether of bisphenol A (DGEBA) with an epoxide equivalent weight of 172–176 g.mol^−1^ and the curing agent 4,4′- methylenedianiline (MDA) were purchased from Aldrich Chemical Co. The ionic liquid 1-butyl-3-methylimidazolium chloride ([BMIM][Cl]) was obtained from Fluka. The chemicals were used as received.

Epoxy resin containing all concentration of IL was studied in our previous research^16^ where it was observed that materials with high level of room temperature flexibility were only achieved at 40% of [BMIM][Cl] in epoxy. An IL content below 40% was found to be more ductile and rigid and at more than 40% of IL content the material started to loose stability and integrity. Epoxy mixtures containing 40 wt% IL were mechanically stirred for 1 hour. For the fabrication of epoxy composites pre-weighed amounts of nanoplatelets were first suspended in the epoxy [BMIM][Cl] mixture and then sonicated (1000 W, 20 KHz) for 1 hour. The resulting suspensions were mechanically stirred for 2 hours and then mixed with MDA. After complete dissolution of the MDA, the whole mixture was poured into pre-heated (100 °C) moulds, cured at 150 °C for 10 hours and then post-cured at 180 °C for 2 hours, with excess ILs were extracted out. Imidazolium based room temperature ILs possess high thermal stability. The decomposition temperature of BMIMCl is 291 °C. Hence the high temperature curing and post curing has no impact on the stability of IL or the nanocomposites.

The small angle x ray scattering (SAXS) beam-line at the Australian Synchrotron was used with a 1.6 m camera length to investigate the scattering patterns following a previously described procedure^1^. The q range was from 0.008 to 0.5 Å^−1^. An in-vacuum undulator source (22 mm period, K_max_ 1.56) at a beam energy of 9 keV was used. The exposure time for each sample was 2 s and tests were performed at 25 °C. The intensity profiles were interpreted from the plot of scattering intensity (I) as a function of scattering vector (q) which is related to the scattering angle θ by the relationship q = (4π/λ) sin (θ/2).

Dynamic mechanical tests were performed on a TA Q800 dynamic mechanical thermal analyzer (DMTA) operating in dual cantilever mode with a frequency of 1 Hz and a heating rate of 3 °C/min. The specimen dimensions were 60 mm × 12 mm × 3 mm. The storage modulus, loss modulus and tan δ were measured from 30 to 250 °C. The *T*_g_ was taken as the maximum of the tan δ curve in the glass transition region.

The tensile properties of the composites were measured on an Instron 30 kN SD tensile testing machine. The tests were conducted using dumb-bell shaped specimens according to ASTM standard D638. A minimum of five specimens were tested for each sample and the average values reported.

DSC experiments were performed under a nitrogen gas atmosphere on a TA-DSC model Q200 instrument using 5–10 mg of each sample. The samples were first heated to and held at 100 °C for 5 min to remove the thermal history. The samples were cooled to −80 °C at a rate of 20 °C/min, held for 5 min, and finally heated from −80 to 250 °C at a rate of 20 °C/min (second scan). The *T*_g_ values were taken as the midpoint of the transition in the second scan of the DSC thermograms.

The electrical resistivity of the samples was determined using an electrical resistivity cell with variable resistor and a distance between the electrode probes set at 1 cm. Prior to testing, the samples were polished for 30 seconds using 1200 grit paper at 150 RPM on a Struers Rotopol-1 polish machine (Struers Australia, QLD).

The morphologies of the nanocomposites were examined using a Zeiss Supra 55VP scanning electron microscope (SEM) operating at an accelerating voltage of 5 kV and a working distance of 10 mm. The cryo-fractured surfaces were coated with thin layers of gold to avoid charging.

Raman spectra were obtained at 1064 nm excitation and a resolution of 4 cm^−1^ using a Bruker RFS-100 FT-Raman spectrometer equipped with an Adlas Nd:YAG laser and a liquid nitrogen cooled Germanium diode detector. The samples were held on a mirrored backing in 180° backscatter geometry. Data acquisition, 256 scans, was performed using Bruker OPUS software (version 3.1). The laser power was 500 mW. A Blackman-Harris 3-term apodization function was used. Data manipulation was carried out using Grams AI software v9.1.

Raman spectra were obtained at 514 and 785 nm excitation using an inVia confocal microscope system (Renishaw, Gloucestershire, UK) through a x50 (0.75 na) objective. Incident laser powers were 0.8 mW (514 nm) and 2.3 mW (785 nm) and a coaxial backscatter geometry was employed. Spectra were collected over the range 100 to 3200 cm^−1^ and averaged over at least 4 scans, each with an accumulation time of 40 s. The Raman shifts were calibrated using the 520 cm^−1^ line of a silicon wafer. The spectral resolution was~1 cm^−1^. Data collection and manipulation was carried out using WiRE 4.2 software.

## Results and Discussion

Thermal flexibility behavior can be induced in epoxy thermosets by introducing ionic liquid charge transfer complexes between functional groups within cross linked epoxy and ionic liquid ions^16^. It has been reported that by varying the epoxy - ionic liquid ratio, materials could be produced that can exhibit distinct physical and mechanical characteristics ranging from those of brittle thermosets, to perfectly ductile thermoplastics, to elastomers. In the present work, a plastic-like thermoset composition was chosen to form the matrix for graphene nanocomposites. This matrix composition was prepared by using epoxy and the ionic liquid 1-butyl-3-methylimidazolium chloride ([BMIM][Cl]) at a 60/40 (w/w) ratio. At room temperature, this cured thermoset composition can be bent and twisted by applying a relatively small force and the polymer retains the new shape even after the force is removed. The room temperature flexibility of these thermoset materials were explained by the confinement of ionic liquid molecules within the highly cross-linked thermoset and the charge triansfer complex formation between the components. Here, with a high ionic liquid content (40%), the bulky ions form a coupling between the epoxy cross links which break and reform when external forces are applied resulting in the flexibility of the material at room temperature.

**Figure 1:**
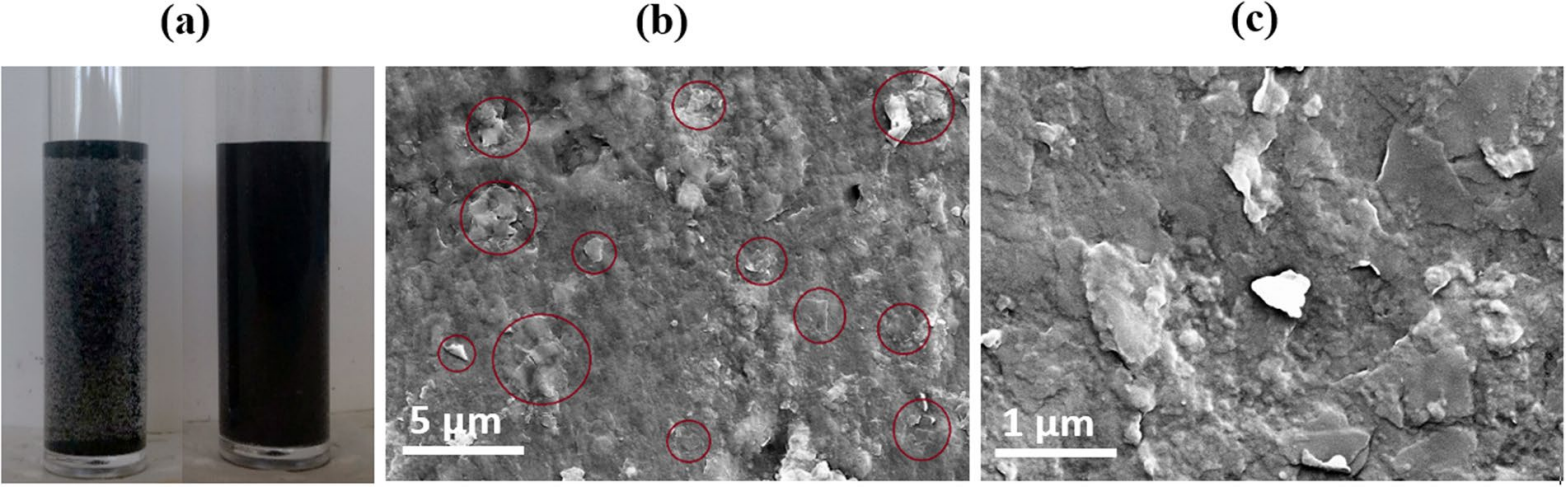
(**a**) photographs of 1 wt% graphene nanoplatelet suspensions in neat epoxy (left) and epoxy/[BMIM][Cl] 60/40 mixture (right), both after 1 hour sonication; SEM images of nanocomposite with 0.6 wt% graphene loading at low (**b**) and high (**c**) magnifications (with platelets shown in red circles).

The non-covalent dispersion of carbon nanomaterials such as carbon nanotubes and graphene has been critical in the preparation of nanocomposites to increase the utilization of these materials while maintaining their inherent reinforcing and electronic properties. Ionic liquids have been investigated extensively in the past and found to be excellent non-covalent dispersing agents for carbon nanotubes (CNTs) in preparing bucky gels^17^ and polymer nanocomposites^18^. The dispersion of individual CNTs and the curing of epoxy, both controlled by ionic liquids, were achieved in our laboratory^18,19^. In the present work, the combination of graphene platelets with ionic liquids leads to a high level of dispersion which was then utilized in making a particular type of room temperature flexible nanocomposites with improved properties. The graphene nanoplatelets were used “as received” without any purification or functionalization knowing that ionic liquids can uniformly disperse graphene. The high level of dispersion of the graphene nanoplatelets with the aid of the IL is clear from Fig. 1a.

When a few layers of graphene are present, splitting of the G mode phonon makes it become active in the infrared spectrum near 1613 cm^−1^ (200 eV). This weak feature would be easily masked by the strong aromatic ring modes of the epoxy. While graphene is inactive and multiple layer of graphene absorb weakly in the infrared, both can be easily detected using Raman spectroscopy. Raman mapping with its high spatial resolution of 0.5 μm between analysis spots has potential to reveal the spatial distribution of the graphene within the epoxy matrix. While excellent Raman spectra were collected from the epoxy and epoxy/IL mixtures using both 785 and 1064 nm excitation, once the graphene was added no useful data could be obtained (not shown). Raman spectra of graphene are easily obtained using visible excitation. The epoxy was found to fluoresce, washing out all Raman spectral features, when 514 nm excitation was utilized (not shown).

The distribution of graphene nanoplatelets in the epoxy/[BMIM][Cl] matrix was first examined using SEM. The SEM images of a graphene epoxy nanocomposite with a 0.6 wt% graphene loading shown in Fig. 1b and c which illustrate that the graphene sheets are randomly aggregated and distributed throughout the matrix phase. The graphene platelets have lateral dimensions ranging from several hundred nanometers to several microns. The nanoplatelets appear to be exfoliated and dispersed evenly in the epoxy matrix as shown by the circled areas in the SEM images. The high level of dispersion of platelets in the epoxy is due to the presence of the ionic liquid that preferentially interacts with individual graphene sheets through π-π stacking interactions^20^.

The composites were further analyses using Raman spectra (not shown). In the infrared-to-visible spectral range, the expected absorbance has been calculated to be independent of frequency and to have a magnitude given by *πα* = 2.293%, where *α* = *e*^2^*ћc* ^21,22^. This means that over this spectral region each graphene layer absorbs 2.3% of the incident light. The optical absorption in graphene is dominated by interband transitions from the mid-infrared to the ultraviolet^21,22^. In this study, the absorption in the near infrared region (>0.8 eV) has been used to precisely determine the number of graphene layers present in sample comprised of 3 to 6 layers^23^.

**Figure 2:**
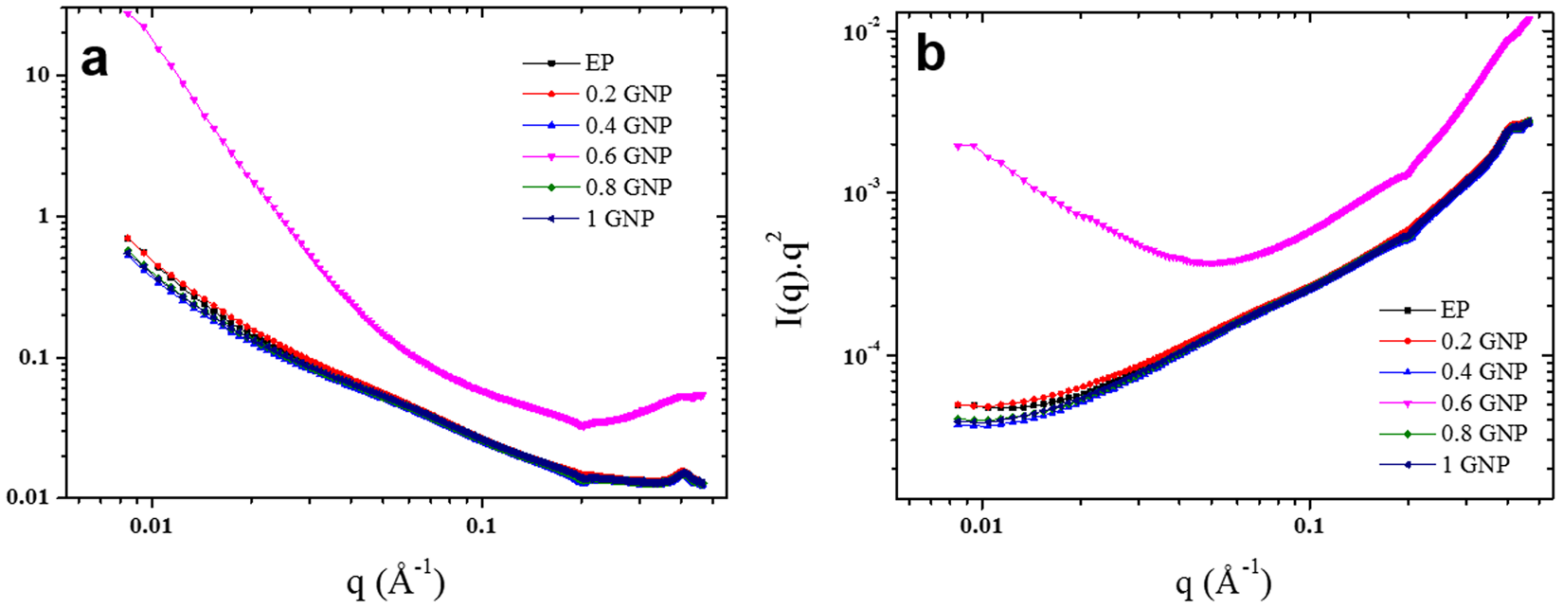
(**a**) SAXS profiles of flexible epoxy nanocomposites with various graphene nanoplatelet loadings, and inset of representative scattering pattern, (**b**) Lorentz plots for the series of samples.

Representative SAXS profiles in terms of intensity as a function of scattering vector q for different graphene loadings are presented in Fig. 2a alongside that of the pristine epoxy resin material. The SAXS scattering intensity was demonstrated to reflect either potential stacking of nano-materials, such as graphene platelets, or specific interactions between nano-materials and macromolecular chains arising from self-assembly or crosslinking^24^. SAXS investigations of the nanocomposites were therefore performed to probe the degree of dispersion of the graphene within the reinforcing matrix. Interestingly, little difference in scattering signals can be observed, even at high graphene loading, over the q range investigated. The Lorentz plots (I(q)q^2^ vs q) shown in Fig. 2b, also confirms that no specific structural characteristic correlation distances could be extrapolated with increasing amounts of graphene. The absence of any diffraction features confirms the excellent dispersion of the graphene nanoplatelets within the matrix and the low density of long range physical interactions between the two materials. Although segregated graphene platelets may be present within the matrix, these results, consistent with previous work reported in the literature, confirms that the addition of filler in the crosslinked epoxy resin generally leads to a low degree of π–π stacking in the graphene^25^, and that the ILs provided a suitable platform to improve graphene dispersability^26^.

**Figure 3:**
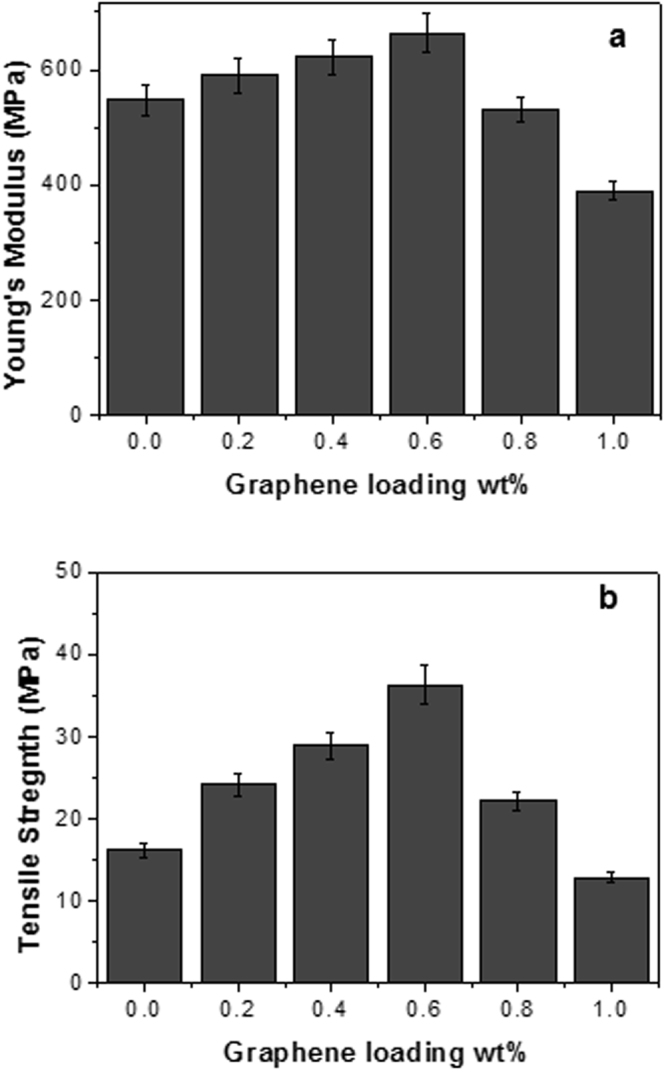
Young’s modulus (**a**) and tensile strength (**b**) determined for flexible epoxy nanocomposites with various graphene nanoplatelet loadings.

Figure 3 shows the variation in tensile mechanical properties of the epoxy matrix and the nanocomposites with different nanoplatelet loadings. It can be seen that the Young’s modulus and tensile strength of the epoxy matrix increased with the addition of graphene nanoplatelets whereas the elongation decreases. The Young’s modulus and tensile strength of the epoxy containing 40% of the ionic liquid are 547.28 MPa and 16.16 MPa respectively. A significant increase of 125% in tensile strength and a moderate increase in Young’s modulus (21%) were observed in nanocomposites with 0.6 wt% graphene loadings. Figure 3 also shows that graphene loadings above 0.6 wt% considerably reduced the mechanical properties of the nanocomposites. The decreased elongation with increasing graphene content observed in these nanocomposites is typical of a composite with enhanced strength and stiffness^27^. It is apparent that graphene nanoplatelets can significantly enhance the mechanical properties of epoxy/ionic liquid flexible thermosets. This mechanical enhancement can be attributed to (i) high inherent elastic modulus and strength of graphene nanoplatelets (ii) better interactions between graphene and the epoxy/[BMIM][Cl] matrix and (iii) a more uniform graphene dispersion in the epoxy due to the presence of ionic liquid.

**Figure 4:**
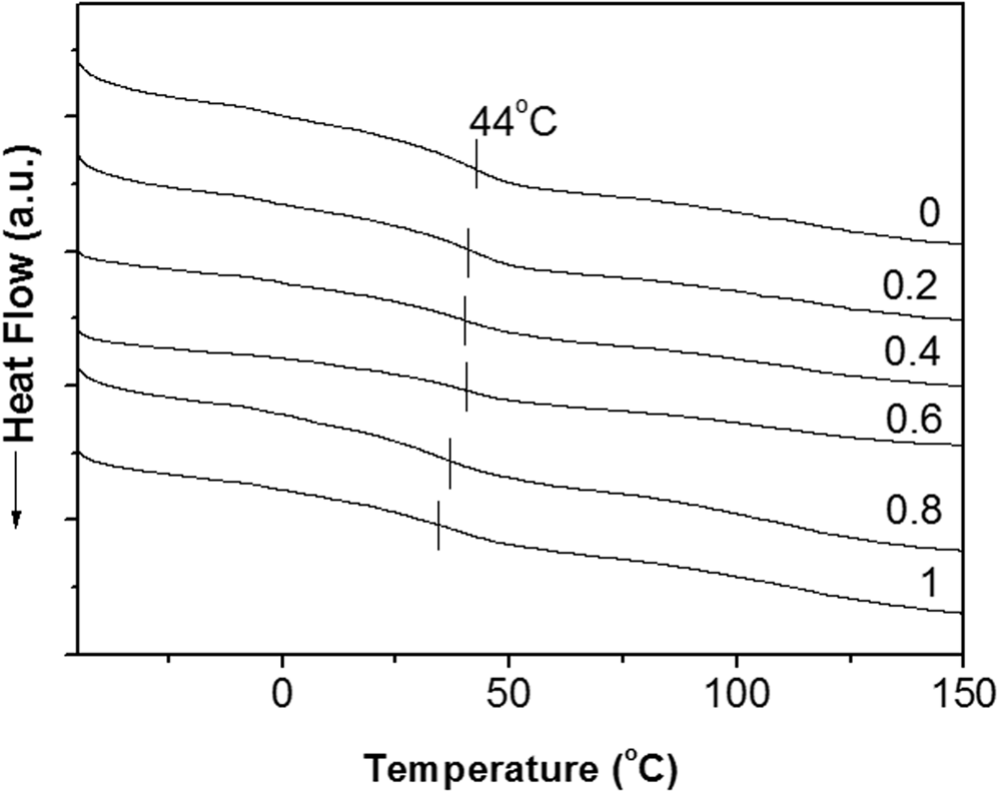
DSC thermograms obtained from flexible epoxy nanocomposites with various graphene nanoplatelet loadings (wt%).

**Figure 5:**
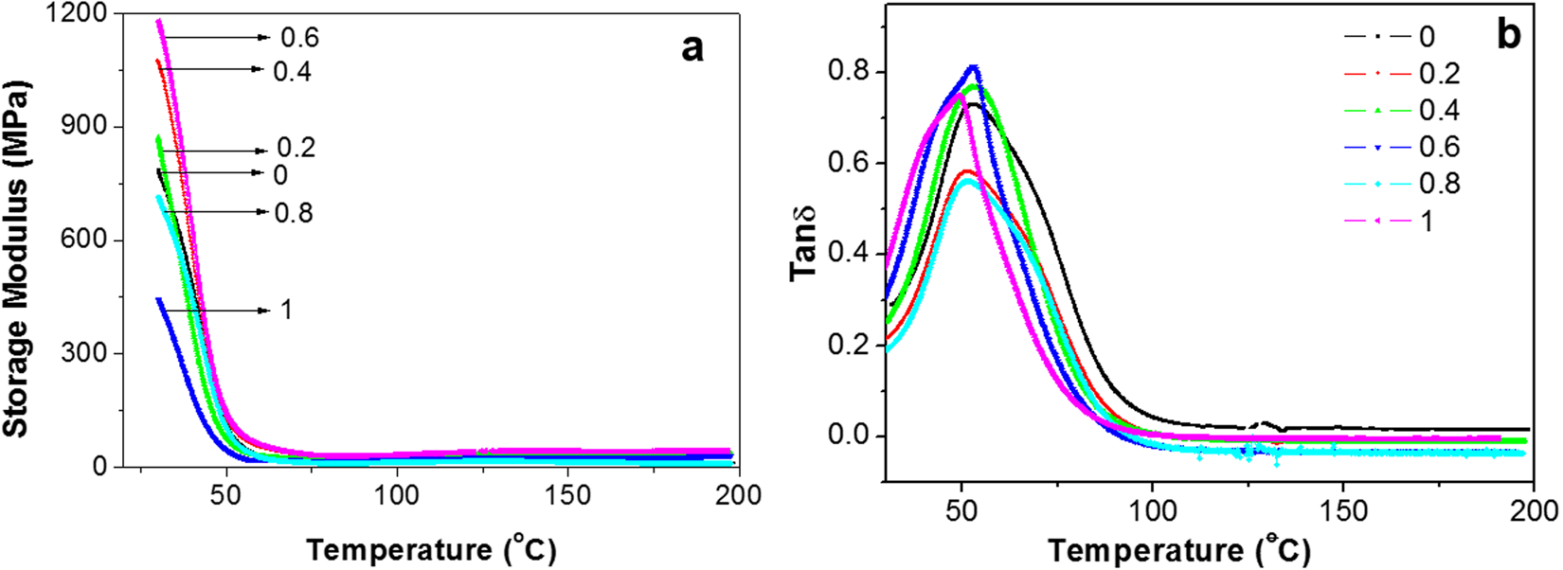
The variation in (**a**) storage modulus and (**b**) tan δ with temperature for flexible epoxy nanocomposites with various graphene nanoplatelet loadings (wt%).

The thermal and thermo-mechanical properties of the nanocomposites were analyzed and the results are shown in Figs 4 and 5. The thermal behavior of the flexible epoxy nanocomposites was investigated. The top DSC curve in Fig. 4 shows that the epoxy thermoset containing 40% [BMIM][Cl] has *T*_g_ of around 44 °C. The *T*_g_ of the flexible epoxy matrix remains about the same with the addition of graphene nanoplatelets and only decreases marginally (~3 °C) at higher (0.8 and above) graphene loading. In the previous research it was found that epoxy containing 40% IL become very soft when heated above *T*_g_. Generally a reduction in *T*_g_ is attributed to either molecular chain confinement by the graphene sheets or the impact of the graphene sheets on the cross linking reactions of the epoxy^4^. In both cases, the polymer cross-link density decreases leading to an increase in polymer chain mobility.

The storage modulus of epoxy was found to improve with the addition of graphene (Fig. 5a). At room temperature, the nanocomposite with 0.6 wt% graphene exhibited a nearly 50% increase in the storage modulus, which is quite significant. The storage modulus reduces above 0.6 wt% graphene content which is consistent with the tensile mechanical properties discussed previously. The glass transition as measured from the tan δ plots (Fig. 5b) also suggests that the *T*_g_ of the epoxy remain more or less unchanged with the addition of graphene.

**Figure 6:**
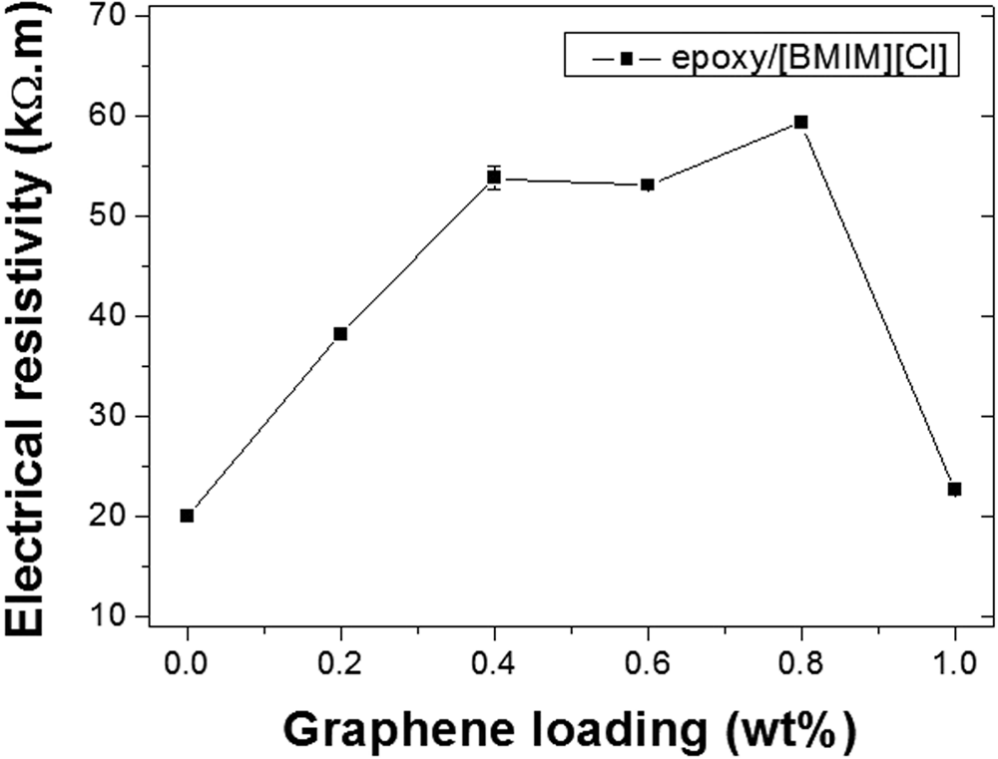
Electrical resistivity of the epoxy nanocomposites with various graphene nanoplatelet loadings.

Interestingly, the electrical conductivity of the graphene IL composites was found to decrease with graphene loading content (Fig. 6). The bare IL – epoxy samples exhibited the highest electrical conductivity, while the pure epoxy resin samples were completely insulating. Although this result was expected from the fact that the concentration of graphene across the structure was too low to allow for the formation of a continuous percolation threshold of graphene, the trend was indicating a level of synergistic interactions between the ILs and the graphene nano-sheets. Indeed, the highest resistance within the graphene IL series, was found to be reached for the 0.6 wt% graphene loading samples which suggests a level of self-organization between the ILs and the graphene sheets in the matrix of the composite. It is likely that the graphene adsorbed part of the ILs by electrostatic interactions in solution, prior to curing of the resin, which may have led to a depletion of the IL concentration and to a less IL conductive network^28^. Such interactions would likely lead to a level of polarization of the structure, which was also experienced when testing the samples, as all the IL graphene composite materials exhibited a capacitor behavior by altering the charge propagation and overall surface charge of the bulk material at the graphene-epoxy interfaces^29^. The stabilization of the current across the samples was slow, and took up to a few minutes for the highest graphene loadings. The use of polymerizable ionic liquid crystals may offer opportunities to further reinforce such matrixes and generate epoxy resin materials into semi-conducting structures with various levels of electronic responses^30^.

**Figure 7:**
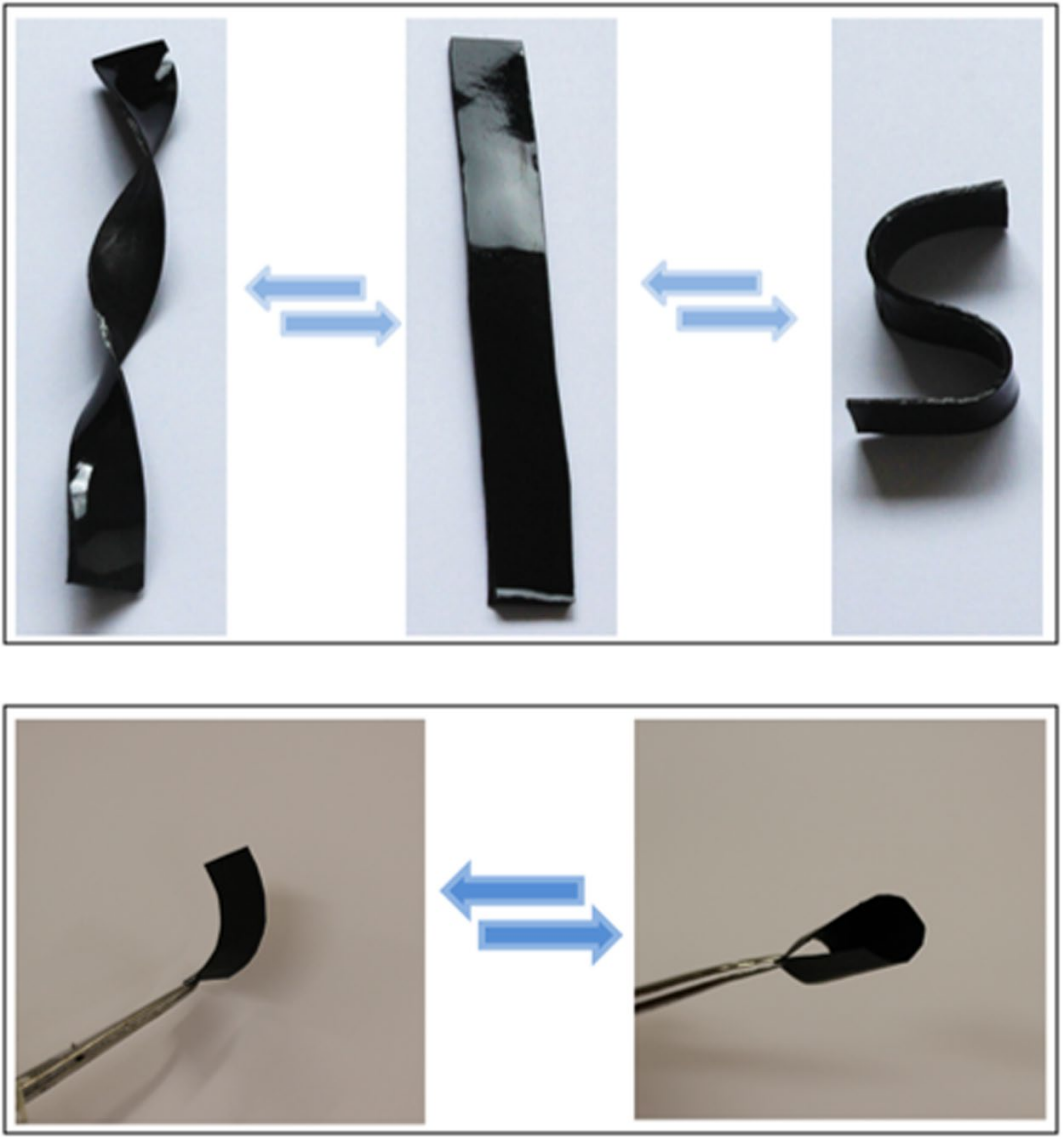
The reversible room temperature flexibility of the nanocomposites demonstrated using thick rectangular specimens (top) and membranes (bottom).

The graphene nanocomposites were all flexible at room temperature. The materials can be reversibly bent or twisted many times by applying a local force. Figure 7 shows thick (2 mm) rectangular and thin (~300 μm) membranes nanocomposite specimens with 0.6 wt% graphene loadings. The rectangular specimen was easily twisted and bent to produce fusilli and curved shapes and the membrane could be folded with no physical deformation observed in these materials.

## Conclusions

While commonly reported for thermoplastics, inducing flexibility into thermosets and fabricating bendable and foldable nanocomposite materials remains a great challenge. This is primarily due to the lack of suitable materials that combine both mechanical flexibility and superior conductivity for their high performance in electronic devices. Room temperature flexible nanocomposites were here prepared for the first time using epoxy and graphene nanoplatelets. An IL was used to induce network flexibility into a cross-linked epoxy thermoset resin. The IL also served as a functional dispersant for graphene nanoplatelets. A morphological investigation showed highly dispersed nanoplatelets in the matrix phase with the high level of dispersion attributable to the interaction between graphene and ionic liquid. The incorporation of graphene significantly improved the tensile mechanical properties of the epoxy with a huge 125% improvement in tensile strength. The storage modulus showed a 50% improvement at a 0.6 wt% graphene loading whereas the thermal properties of epoxy were not affected by the incorporation of the graphene. The nanocomposites and films show high levels of flexibility at room temperature where it almost behaved like a thermoplastic. It has been identified that the strong interaction between bulky IL ions and the epoxy hydroxyl groups link the brittle epoxy chains network. During physical deformation (or when heated), such secondary bonds reversibly break leading to flexible cross-linked networks. The electrochemical properties have to be further investigated to explore the potential of these materials in flexible electronic devices.
